# Reaching Forest Workers with Yellow Fever Vaccine Through Engagement of the Private Sector in Central African Republic

**DOI:** 10.3390/vaccines12121424

**Published:** 2024-12-17

**Authors:** Gertrude Noufack, Placide Bissengue, Junior Koma Zobanga, Junior Stève Cyrille Malingao, Mory Keita, Marie Constance Razaiarimanga, Marie-Eve Raguenaud

**Affiliations:** 1 World Health Organization Regional Office for Africa, 106 Djoue, Brazzaville P.O. Box 06, Congo; noufackg@icloud.com (G.N.);; 2 Ministère de la Santé et de la Population de la République Centrafricaine, Bangui, Central African Republic; 3 World Health Organization Country Office, Avenue Gamal Abdel Nasser, Bangui BP 1416, Central African Republicrazaiarimangam@who.int (M.C.R.); 4 Institute of Global Health, Faculty of Medicine, University of Geneva, 1211 Geneva, Switzerland; 5 World Health Organization, 20 Avenue Appia, 1211 Geneva, Switzerland

**Keywords:** yellow fever, high-risk workers, epidemic, public–private partnership, vaccination campaign

## Abstract

**Background/Objectives**: Yellow fever (YF) outbreaks continue to affect populations that are not reached by routine immunization services, such as workers at a high risk of occupational exposure to YF. In the Central African Republic (CAR), YF cases were detected in districts characterized by the presence of workers in forest areas. We developed an innovative approach based on a local partnership with private companies of the extractive industry to administer YF vaccine to workers in remote areas during the response to an outbreak. **Methods**: The planning stage of the campaign included the mapping of forestry and mining companies through the involvement of national and/or local representatives of companies from both the formal and informal sectors. Information sessions and mobilization targeted the heads of operating companies. Advanced and mobile strategies were used to target workers on their work site. Companies provided logistical support including transportation and communication and set up temporary vaccination posts. **Results**: Using this local partnership, it was possible to vaccinate over 70,000 workers (5.8% of the entire vaccinated population) in hard-to-reach areas, protecting them from YF. This represented around 47% of the estimated number of workers and dependents. The partnership with the private sector also contributed to increasing knowledge on the risk of YF and means of protection among a high-risk community. **Conclusions**: Private companies represent potentially useful actors that can contribute to the protection of high-risk workers and to the prevention and control YF outbreaks. The experience in the CAR has demonstrated that it is possible to obtain support from private companies, including informal ones, for a vaccination campaign.

## 1. Introduction

Yellow fever (YF) is an acute viral vaccine-preventable and vector-borne disease in tropical regions of Africa and Latin America [1]. There is no specific treatment for YF and the case fatality of severe infections ranges from 31 to 47% [2]. In 2018, it was estimated that about 109,000 severe infections and 51,000 deaths occurred annually [3]. The YF vaccine is considered the main preventive measure and component of the public health response to an outbreak of YF. The Eliminate Yellow Fever Epidemics (EYE) Strategy by the World Health Organization (WHO), GAVI, and UNICEF emphasizes the importance of vaccination as a primary tool to protect at-risk populations and prevent international spread [1]. A single dose of the YF vaccine provides life-long protection [4].

YF poses a significant public health threat in Africa, where a re-emergence of outbreaks was observed since 2020 [5]. The Central African Republic (CAR) is mapped by the WHO as at a high risk of YF [1]. The country has reported multiple outbreaks of YF in recent years and the highest caseload in Africa in 2022 [3,6,7]. YF is transmitted in tropical forests between non-human primates and mosquitoes. Humans entering these areas can contract the virus through infected mosquito bites, eventually triggering rural or urban outbreaks when returning from the jungle [8]. The urban cycle, where humans are the primary host and *Ae aegypti* is the vector, is a major cause of concern because it results in large-scale epidemics [8,9]. In high-risk countries, workers in a wide range of industries (such as the oil and mining industries, construction, and forestry) are particularly exposed to sylvatic transmission when their activities take place in forests or recently deforested areas. In the CAR, pygmies are also highly exposed to infected wild mosquito bites because they live in forests. Nomadic cattle herders, who spend time at the forest edges, are also at a high risk due to their proximity to forest mosquitoes. It is unlikely that workers in tropical forests especially of the informal sector protect themselves efficiently against mosquito bites [10,11]. Also, it is difficult to reduce mosquito populations in forest areas. Vaccination is thus considered the best way to prevent YF in forest workers and the EYE Strategy recommends the development of strategies to ensure the protection of all high-risk workers [1]. Vaccinating these workers not only safeguards their health but also reduces the risk of YF spreading to other regions, including urban areas, and countries.

Despite past preventive mass vaccination campaigns in 2010, reaching around 90% of the general population aged 9 months to 60 years, as well as reactive vaccination campaigns in selected districts in 2022 in response to the detection of two YF outbreaks [7], six confirmed cases of YF were detected in 2023 in five districts of the CAR (Mbaiki, Berberati, Bossembele, Sanga-Mbaere, and an internally displaced population (IDP) camp in Bambari) [12]. These districts are characterized by an underperforming routine immunization program and the presence of vulnerable groups often missed by routine immunization, such as those living in hard-to-reach areas, IDPs, returnees, and forest workers. The security situation in the country remains precarious due to recurring socio-political unrest following military conflicts in 2013. This situation has led to poverty, economic instability, and the forced displacement of populations, making it difficult for them to access basic social services [13].

Migrant and seasonal workers, especially in the informal sector, are particularly difficult to reach during routine vaccination activities or standard vaccination campaign due to the absence of census data and adapted vaccination strategies. Existing research on strategies to protect individuals from exposure to vector-borne diseases has been found to be biased towards tourism and the military [14], while attention and resources should target vulnerable populations most exposed and in need of protection against yellow fever, like forest workers including migrant workers, in endemic countries. Studies in sub-Sahara African countries on public–private partnerships to strengthen vaccination often focus on childhood immunization [15,16].

In the CAR, recent discoveries of gold have fueled the development of artisanal mines and attracted an informal work force and buyers from neighboring countries. Porous borders with the Democratic Republic of the Congo and the Republic of the Congo have facilitated cross-border population movements. Other economic activities, such as caterpillar breeding, increase population movements into forest areas during the rainy season each year (seasonal workers).

Due to the risk of further spread of the epidemic, health authorities decided to conduct a reactive vaccination campaign targeting the general population in the five affected districts and two neighboring districts, taking due consideration of the unprotected population of workers with a high risk of exposure. In mining areas of the DRC characterized by mobile populations, the national EPI uses multiple vaccination strategies to target hard-to-reach children (GAVI.org website (accessed 16 December 2024)). Similarly, an adaptation of the vaccination strategy was needed to effectively reach high-risk workers in the CAR. This article describes a novel approach that the national health authorities of the CAR developed to reach forest workers to administer a dose of the YF vaccine during a reactive vaccination campaign (RVC) to prevent the propagation of a yellow fever epidemic.

## 2. Materials and Methods

**Figure 1:**
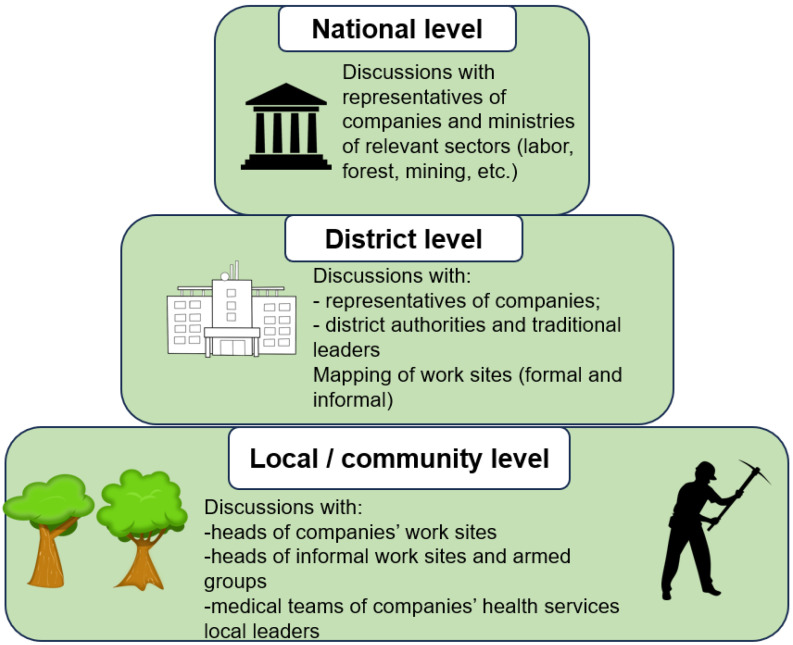
Process to engage with private partners for a reactive vaccination campaign, Central African Republic, 2023–2024.

In parallel to the activities implemented to prepare for the reactive vaccination campaign targeting the general population, specific initiatives were taken to target high-risk workers (e.g., seasonal workers, miners, fishermen), including pygmies living and working mostly in the forest. Before organizing the vaccination campaign, it was essential to engage with representatives of the private sector, both formal and informal, in all key preparatory stages. The overall process to engage with private partners required interactions at the national and subnational levels (Figure 1).

### 2.1. Planning

The strategy to vaccinate forest workers was developed during central level coordination meetings led by national health authorities, including Expanded Program on Immunization (EPI) managers, and attended by public health officers, United Nations technical agencies such as the World Health Organization (WHO) and the United Nations Children’s Fund (UNICEF), and other partners such as the Global Alliance for Vaccines and Immunization (GAVI) and the Agence Européenne pour le Développement et la Santé (AEDES).

The implementation planning of the workers’ vaccination was integrated into the general outbreak response plan and micro-planning. A national micro-planning workshop was organized at the Ministry of Health with the presence of regional directions and the seven health district management teams and partners. One of the outputs was a spreadsheet tool used to facilitate the mapping of the groups of workers and the recording of an initial estimate of the number of forest workers to be vaccinated. The national ministries of forest (Ministère des Eaux Forêts, Chasse et Pêche) and mines (Ministère des Mines et Géologie) provided information on the number and location of mining sites and forest industries, including the number of employees in each officially registered company. Outreach to regional representatives of the ministries was necessary to complete and consolidate this information. District health authorities then reached out to local representatives of companies to inform of the planned vaccination campaign and to obtain updated counts on workers present in the districts. As there was no listing of informal work sites available at the national level, the mapping was further completed by the health district authorities who convened local community leaders at meetings. While local leaders were able to provide the locations of informal work sites, local heads of these sites were contacted to provide the number of workers and their families based on the local census. Community leaders were informed before the district health team visited mining sites in security-compromised areas.

### 2.2. Materials and Equipment

A vaccinator’s guide and training modules were made available to health districts, to guide them on vaccination strategies, the vaccine administration method, the surveillance and management of adverse events following immunization, the management of vaccines and devices, and the management of vaccine wastage. Tally sheets and the database were adapted to capture vaccination information on the different types of workers. Transport logistics and cold chain equipment were provided by the public district health authorities and a few private companies.

### 2.3. Training

Health personnel from private companies, especially from forest companies, participated in training sessions organized in two districts. Local mobilizers, essentially workers and traditional leaders, were trained on communication and social mobilization prior to the vaccination campaign. Health personnel from the medical services of several companies and district health personal received refresher training one day before the vaccination started.

### 2.4. Communication

Specific communication targeting local representatives of companies in Berberati and Bossembele districts was conducted before the reactive vaccination campaign. An information session for traditional and religious leaders, local economic operators, and other district actors took place prior to the campaign. Information sessions were held with several company managers. Separate sessions were held with local armed groups controlling several mining sites to facilitate and safeguard access for vaccination teams in several mining sites. A press release with information on YF clinical presentations, modes of transmission, protective measures, the adverse events of vaccination, and the campaign’s date and duration, was released and shared with political leaders, religious and traditional leaders, and company and site managers. Banners and posters were distributed in each commune and company workplace. At the regional level, social mobilization meetings were attended by health officials, local authorities/leaders, forestry and mining leaders, traditional leaders, and heads of informal exploitation sites (not managed by a private company). These meetings aimed to facilitate access, raise awareness, and organize vaccination in geographically and security-challenged areas. Social mobilization also provided an opportunity for discussions with the various local authorities, especially those responsible for forest worker sites and heads of operating companies.

### 2.5. Vaccination Campaign Implementation

The first reactive vaccination campaign targeted the population of Mbaiki district and took place from 27 September to 2 October 2023. The second campaign was implemented from 6 to 10 July 2024 in six other health districts. To reach remote work sites, the vaccination strategy relied on advanced teams (four members on the vaccination site set up for one or two days) and mobile teams (three members using motorcycles to reach mobile populations and small remote sites). Temporary mobile teams were deployed in forest camps, logging sites, and miners’ sites. Mop-up vaccination activities were conducted on the companies’ work premises or settlements, with support from personnel of the companies after the first vaccination campaign to increase coverage. Forestry companies provided logistical support, including transportation, temporary medical facilities, and communication support using their telephonic communication system, during the second phase of the campaign. For example, the cold chain equipment was kept in the compound of the largest mining company in Bossembele district.

### 2.6. Coordination and Supervision

The same local operational coordination team from health centers supervised all vaccination activities including the ones targeting forest workers. Feedback was provided to workers and managers.

A post-vaccination campaign national workshop to identify lessons learned was held in Bangui on 26 of July in the presence of two government ministers.

Vaccines were provided free of cost through the mechanism of the International Coordinating Group (ICG) on Vaccine Provision. As a GAVI-eligible country, the CAR received financial support to cover the operational cost of the campaigns.

## 3. Results

A total of 1,209,085 people were vaccinated during the reactive vaccination campaigns against yellow fever in the districts of Mbaiki, Boda, Bossembele, Berberati, Carnot-Gadzi, Gamboula, and Sangha-Mbaere. Among them, 70,493 (5.8%) were workers and Pygmies and their dependents (Table 1). The proportion of vaccinated workers and Pygmies among all vaccinated individuals ranged from 1% in Boda district to 23% in Gamboula district. Workers were present in all seven districts where yellow fever cases had been reported. Pygmies were included as they live and work in forests and represented 36% of all workers reached during the reactive vaccination campaign. Workers and pygmies targeted by the campaign were identified from 97 geographical sites (Table 2).

Based on the figures from the mapping of workers carried out to plan the vaccination campaign, the vaccination coverage rate of high-risk workers is estimated to the 47%. This is below the 80% threshold necessary to the interrupt local transmission of the YF virus within a community and to ensure that sporadic unvaccinated cases do not generate additional cases [1]. The vaccination coverage of workers including pygmies per district ranged from 12% in Sangha Mbaere to 94% in Boda (Table 1). Based on the estimates of total workers, workers in the informal sector represented a higher proportion (77%) than workers from the formal sector. Security-compromised work sites were all informal work sites. Health authorities had to negotiate with armed groups controlling work areas ahead of the vaccination campaign, to ensure that mobile teams could safely access workers. The negotiations between some army units and local health authorities enabled the implementation of vaccination activities in the security-challenged areas of the Bossembele, Gamboula, and Sangha Mbaere districts.

**Table 2:** Estimates the number of high-risk workers including forest workers, miners, Pygmies, and fishermen, in seven health districts of the Central African Republic used for planning the reactive vaccination campaigns.

District	Workers of the Formal Sector	Communities of the Informal Sector	Number of Workers and Dependents	Number of Work Sites	Issues with Access
Mbaiki		Pygmies	25,120	20	Hard-to-reach access areas. Accessible by river only
		Fishermen	230		
		Miners	200		
	Seasonal forest workers		1190	1	
Boda		Pygmies, workers	1809	6	
Bossembele		Fishermen’s Camp	1187	11	Compromised security zone
		Mining sites	30,249		
Gamboula	Seasonal forest workers		1100	3	
	Fishing colonies and mining cooperative		12,700	3	River sector, hard to reach
		Miners	23,145	21	Insecurity (presence of armed groups)
Berberati		Miners, Pygmies Fishermen	18,432	15	High population concentration Insecurity in three zones
	Seasonal forest workers		5179	1	
Sangha-Mbaere		Pygmies, miners, fishermen	12,516	6	
	Seasonal forest workers		10,018	1	
Carnot—Gadzi		Pygmies, fishermen	6870	9	High mobility of population during the dry season.
Total			149,945	97	

A total of 66 mobile and advanced teams were involved in the deployment of 132 vaccinators and 132 volunteers, the majority of whom came from the public health district and facilities. A total of 97 work sites were reached by the vaccination teams. The vaccination teams had mixed personnel from both the local public health facilities and the health services of private companies. Two private companies provided vaccinators and volunteers from their health centers, including workers and health personnel. Additionally, 66 mobilizers and 66 criers were recruited at each site, comprising workers’ families and traditional leaders of the mining site. Community engagement was also established with local civic organizations, such as women’s associations and community-based associations. All team members were supervised by public health district supervisors.

The mobilization of sawmill operators and the local hierarchy of miners before and during the vaccination campaign contributed to identifying workers in hard-to-reach forest areas due to geographical barriers and insecurity, and their vaccination against YF. Mop-up vaccination activities that aimed to increase vaccination coverage after the main vaccination campaign were held on the companies’ work premises or mining/logging sites. The ad hoc partnership with the private sector allowed the involvement of health personnel of companies in social mobilization and vaccination activities. Their participation contributed to increasing the demand for vaccination by the workers and their families. Forestry companies also provided logistical support, including transportation, temporary medical facilities, and communication systems during the second phase of the campaign. The CAR, being a low-resource conflict-torn country, has infrastructure gaps in many districts and villages. The cold chain equipment used for vaccine storage in Bossembele district was installed in the compound of a private company in Gaga commune.

## 4. Discussion

We found that partnering with private companies operating in the forest environment facilitated access to workers from both the formal and informal sectors in hard-to-reach and insecure areas of the CAR. The low vaccination rate may be attributed to three contributing factors. Vaccination teams were not able to reach all work sites due to insecurity and a lack of geographic access. The low vaccination uptake among informal sector workers may be attributed to their fear and lack of trust in authorities, leading them to avoid contact with district health teams. In the CAR, many seasonal workers are cross-border migrants who may have feared controls and arrest. Another potential explanation for the low coverage is the inaccuracy of the denominator, particularly regarding the number of workers and their dependents in the informal sector, as health authorities relied on estimates provided by work site heads.

Despite the reassuring absence of newly reported cases of YF following the vaccination campaign, non-vaccinated workers exposed to the forest environment remain at risk due to the ongoing virus transmission between non-human primates and mosquitoes. High-risk workers constituted 5.7% of the vaccinated population. Despite this small proportion, their heightened exposure to the YFV and the subsequent potential to initiate outbreaks upon leaving forested areas underscore the critical need to target this special population group for vaccination. Vaccinating workers is crucial for both individual protection and the prevention of the national and international spread of YF.

This is the first time that health authorities in the CAR reached out to the private sector for support in conducting a mass vaccination campaign. In terms of direct support, the benefits were highest during the second phase of the vaccination campaign, particularly during the mop-up, when logistical support was provided by the private companies and vaccination posts were set up on work sites. Allowing workers to be vaccinated at their workplace can increase vaccine uptake. A systematic review reports that studies, although mainly conducted for influenza, have shown that offering free on-site vaccination can be a successful tool to ensure adherence to vaccination campaigns and the administration of required vaccine doses [17]. In low-resource countries like the CAR, where occupational physicians are scarce or nonexistent, workplace vaccination is likely to continue to rely on district health personnel and necessitate preestablished agreement between health authorities and private work sites, from both the formal and informal sectors. Only large companies can be asked to make their medical teams available to support vaccination activities.

The post-campaign evaluation workshop enabled the country to draw lessons with representatives from other involved ministries, thereby strengthening their involvement in future activities, especially in the identification of workers from the formal sector. One main challenge was obtaining an accurate estimation of the number of workers, especially those from the informal work sector. In the CAR, for informal sites, health authorities relied on numbers provided by heads of campsites. To avoid a stock-out of material and vaccines and missed vaccination opportunities, solutions adopted in the CAR were to adopt a large estimate as well as to conduct a mop-up vaccination campaign following intense communication efforts. To improve the accuracy of estimating the number of informal workers, it is essential to explore future methods such as community-based enumeration, geographic information systems, and digital tools for field use.

Another challenge in the CAR was the need for vaccination teams to negotiate with local armed groups controlling informal mining settlements to guarantee safe access to workers and their families. In such security-compromised settings, gaining trust from local stakeholders hinges on targeted communication and transparency, ensuring that all parties are well-informed and feel secure in the collaborative process. One area of concern was that several informal mining sites and companies were reluctant to get involved in a vaccination campaign. This was probably due to fear of sharing information on their employees and activities with authorities. Although collaborating with private companies for public health initiatives can be highly beneficial, potential challenges such as a reluctance to share employee information must be carefully managed. Although effective communication is crucial before undertaking a vaccination campaign [18], in the CAR, administrative personnel from private companies were not systematically informed and insufficiently involved during the vaccination campaign. The shortage of time due to the context of an epidemic response did not allow the conduct of a community rapid assessment to gather key data from the community of workers, which could have helped design tailored community-informed solutions. Among the lessons learned is the need for a well-coordinated information campaign by health authorities at all levels. This requires health authorities to work closely with non-health sectors such as other relevant ministries and representatives of companies at all levels from the national to the local level. Interventions need to be tailored to forest workers and their employers to address their specific needs and ensure their acceptance and create demand for the vaccine. This can be done preferably before an epidemic and before the district-level planning phase, to maximize buy-in by local company representatives. Exploring the use of local workers’ unions to disseminate information on YF disease and vaccine protection can be beneficial, as workers are more likely to trust these unions. The option of a financial incentive to improve vaccination uptake was not considered here as it is not applied in the CAR and there was no evidence of its effect in this context.

The CAR experience has demonstrated that it is possible to protect hard-to-reach mobile populations such as seasonal workers from both the formal and informal sectors through engagement with local representatives of companies and the workers’ leaders. A scoping review on private sector engagement for immunization programs in low-income and middle-income countries suggests that factors that motivate private providers need better consideration in policy and planning [19]. There are incentives that can encourage private companies to support public health initiatives such as direct employee benefits and contributions towards corporate social responsibility credits. There is numerous published evidence describing strategies to facilitate stronger immunization program engagement with the private sector [19,20]. Projects in resource-limited settings underscore the important role that the private sector can bring to achieving immunization goals, especially among underserved populations, and can provide a model for successful public–private partnerships [15,21,22]. In certain contexts, a partnership with the military could even be considered, as was done in Angola for polio vaccination campaigns [23]. However, the term ‘private sector’ often refers to health care providers outside the public sector, rather than non-health private companies.

The CAR experience could represent a starting point for policy makers and stakeholders to develop and implement new workplace prevention strategies for yellow fever vaccination in remote forest areas far from health services to further increase vaccination coverage. This can be achieved by setting up temporary vaccination posts in forest areas or deploying mobile teams to locations near informal work and living settlements. Establishing a partnership with the general management of private companies involved in forest activities, as well as with their local representatives before an epidemic, could improve and facilitate the targeting of workers during vaccination campaigns. Setting up vaccination posts on work premises will increase vaccine uptake by facilitating workers’ access to vaccines.

YF-endemic countries are encouraged to plan for and include high-risk workers in their preventive, reactive, and targeted vaccination campaigns. These efforts could have a greater impact through formal engagement with private companies in the mining, agricultural, and forest industries operating in YF-endemic areas to ensure the protection of all their workers.

To ensure sustainable protection for workers, YF-endemic countries are encouraged to adopt policies mandating free YF vaccination for all high-risk workers. This decision should be just, fair, and non-discriminatory, involving the input of the workers. National occupational health policy should include YF vaccination for high-risk workers. Private companies should be encouraged to protect their workers and families by providing YF vaccination. Other strategies should be explored that prioritize education, access, addressing vaccine hesitancy, and complementary workplace safety measures.

Protecting high-risk workers from YF is a critical component of the global effort to eliminate the disease. In the era of global health, special attention must be paid to these high-risk workers in direct contact with a YF-prone ecosystem and away from health care services. High YF vaccine coverage can be obtained [24] and should be obtained in all endemic areas.

## 5. Conclusions

In conclusion, private companies represent potentially useful actors that can contribute to the protection of high-risk workers and other activities to control YF outbreaks in high-risk countries. The experience in the Central African Republic has demonstrated that it is possible to obtain support from private work entities, including informal ones, for a vaccination campaign. Effective communication at all levels, from the national to the local, involving representatives of companies and informal campsites was crucial for characterizing the workers’ population and ensuring the participation in, and acceptance of, the vaccination campaign. To enhance the impact of private companies on YF prevention, actions such as integrating vaccination into occupational health policies and establishing formal partnerships with key industries are necessary. By working together, governments, private companies, and international organizations can achieve the goal of a world free from YF epidemics.

## Figures and Tables

**Table 1:** Number of vaccinated high-risk workers in seven health districts of the Central African Republic, including forest workers, miners, fishermen, and Pygmies, 2023–2024.

District	Number of Targeted Pygmies and Workers	Number of Vaccinated Pygmies	Number of Vaccinated Miners, Forest Workers, and Fishermen	Total Pygmies and Workers and Dependents Vaccinated	Vaccination Overage Rate of Pygmies and Workers (%)	Total Number of Vaccinated Individuals (General Population and Workers)
Mbaiki	26,740	18,832	996	19,828	74%	218,913
Boda	1809	582	1113	1695	94%	116,213
Bossembele	31,436	NA	12,498	12,498	40%	184,916
Berberati	23,611	1411	5058	6469	27%	200,295
Carnot Gadzi	6870	2986	1653	4639	68%	248,493
Gamboula	36,945	398	22,249	22,647	61%	100,330
Sangha Mbaere	22,534	1356	1361	2717	12%	139,925
Total	149,945	25,565	44,928	70,493	47.0%	1,209,085

## Data Availability

The original contributions presented in this study are included in the article. Further inquiries can be directed to the corresponding author.
